# Changes of Blood Flux at BL21 and Points along BL Meridian Resulted from Acupuncture or Moxibustion: Case Cross Design Study

**DOI:** 10.1155/2017/8237580

**Published:** 2017-07-24

**Authors:** Guangjun Wang, Shuyong Jia, Hongyan Li, Ze Wang, Yuying Tian, Weibo Zhang

**Affiliations:** Institute of Acupuncture and Moxibustion, China Academy of Chinese Medical Sciences, Beijing, China

## Abstract

Acupuncture (Acup) and moxibustion (Moxi) are commonly used interventions in clinical practice. However, the difference between Acup and moxibustion mechanisms is unclear. In current study, blood perfusion responses resulted from Acup or Moxi at Weishu acupoint (BL21) and control points were explored, respectively. The time series of blood flux signals at BL21 and control points were transformed with Morlet wavelet, and the differences in each frequency interval were observed. The results suggested that acupoint response to different stimulation is a comprehensive process which related to all components of blood perfusion signals. Whereas the different response at control points was not observed, there has been significant difference coherence value between Acup and Moxi stimulation. The results suggested the influence of Acup and Moxi not only on the level of blood perfusion at local area; the intrinsic relevance after stimulation which can be evaluated by coherence analysis is also an appropriate index to distinguish different stimulations.

## 1. Background

Acup has been widely used to treat diseases at least for 2500 years [1]. As a type of mechanical stimulation, Acup works by penetrating the skin with needles that are manipulated by the hands or electrostimulation, while Moxi produced thermal stimulation effects by burning moxa. Although Acup and Moxi are different in stimulation, they are believed to have similar clinical outcomes. However, recent study indicated that Moxi and Electroacupuncture (EA) have distinct effect on patients suffering with special type IBS [2, 3]. Further findings suggested that EA and Moxi regulated different network within the brain [4]. The basic research also supported that Acup and Moxi have different effects [5]. In Acup practice, the clinical outcomes generally resulted from the cascade enlargement of local stimulus. Since the efficacy of Acup and Moxi is different, we suspect that distinct stimulation resulted in the different responses at regional acupoint.

Previous studies have shown that acupoint areas are enriched in nerve endings, blood vessels, and mast cells, and these components constitute the basic structure of axon reflex [6–8]. When an Acup point was stimulated, axonal reflex was aroused; then the Acup signal was amplified and transmitted. In this process, the release of vasoactive substances plays an important role, which control the regulation of blood flux. Therefore, regional functional activity can be explored indirectly by observation of local blood flow. Based on the above understanding, we believed that acupoint was aroused by Acup and Moxi in different patterns, which lead to different blood flux in acupoint. The aim of this study is to analyze the difference between Acup and Moxi by detecting changes of local blood flow.

## 2. Methods

### 2.1. Participants

A total of 10 healthy subjects were recruited to the study. The general characters are presented in Table 1. Subjects had to be healthy and aged from 18 to 60 years. All participants were requested to avoid alcohol, tea, or coffee at least 24 hours prior to the test. None of the subjects had any diseases or were taking any medication affecting cardiovascular or autonomic function.

### 2.2. Ethics Approval and Consent to Participate

This study was approved by the Institutional Research Ethics Boards of Acup & Moxi, China Academy of Chinese Medical Sciences. In accordance with the Declaration of Helsinki, each subject provided informed consent and had an adequate understanding of the procedure and purpose of this study.

### 2.3. Protocol for Measurement of Blood Perfusion

Some studies suggested that the meridian system may contain a continuous channel [9] to facilitate signal transport in peripheral tissues [10, 11]. According to this view, when an acupoint which belongs to a special meridian was stimulated, the other part of this meridian will have the corresponding changes. So in the present study we also selected the upper and down points which belong to the bladder meridian (BL) as the control points.

Weishu acupoints (BL21), upper point, and down point were marked by senior Acup doctor. BL21 is in the upper back region, at the same level as the inferior border of spinous process of the 12th thoracic vertebra (T12), 1.5 B-cun lateral to the posterior median line [12]. The upper point is also in the upper back region, at the same level as the inferior border of spinous process of the 8th thoracic vertebra (T8). The down point is in the lumbar back region, at the same level as the inferior border of spinous process of the 4th lumbar vertebra (L4). Both upper point and down point are located at the BL meridian, 1.5 B-cun lateral to the posterior median line.

**Figure 1:**
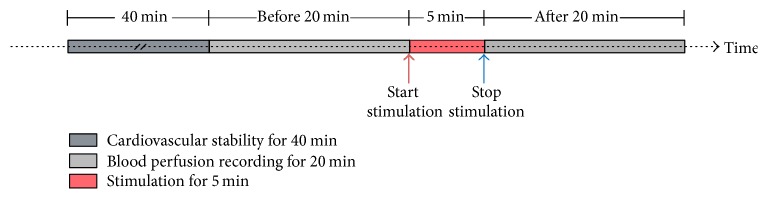
Stimulation schematic diagram.

In the present study, a commercially available FLPI system (moor Instruments, Devon, United Kingdom) was used to measure blood flux at BL21. Before and after stimulation, respectively, as shown in Figure 1, a total of 20 min recordings are continuously acquired at a rate of 25 frames per second with an exposure time of 8.3 ms.

Blood perfusion signals of upper and down point were recorded with PeriFlux System 5000 (Perimed AB, Stockholm, Sweden) at 32 Hz sample rate and 0.2 s time constant. An optical fiber probe connected with the PeriFlux 5000 was used to illuminate and collect the scattered light from the skin tissue. The probe was attached to the surface of interest by a two-sided adhesive tape (PF 105-3, Perimed AB, Stockholm, Sweden). In this study, upper point was recorded by the first channel and the down point was recorded by the second channel. In the current study, all records were performed at the same time.

All measurements were carried out in a quiet, temperature controlled (24–26°C) laboratory. On arrival to the laboratory, subjects were asked to empty their bladders. The participant was asked to assume a comfortable supine position and the skin of BL21 was sterilized. Following a period for cardiovascular stability (40 min), a baseline recording of blood flux was made for 20 min (Figure 1). After baseline recording, the test subjects were stimulated by manual Acup or Moxi randomly; then skin blood flux of BL21, upper point, and down point was monitored for 20 min.

### 2.4. Protocol for Stimulation

For Acup, after baseline recording, a small Acup needle (0.25 × 25 mm, Suzhou Dongbang Acup Inc., Suzhou, China) was gently inserted in a depth of 15 mm in the BL21 acupoint. Before stopping Acup intervention, the needle was slowly rotated for 5 min to maintain the soreness and numbness sensation of De-Qi. For Moxi intervention, after baseline recording, the ignited moxa roll was held approximately 2-3 cm above the BL21, which produced a mild warm and comfortable sensation. Moxi at BL21 lasted for 5 minutes.

### 2.5. Blood Flux Analysis

The recording file of upper and down point was opened in the software of PeriSoft for Windows (version 2.5.5, Perimed, Sweden). The detailed data were exported as txt format and saved. The recording file of BL21 was opened in the software of moorFLPI full-field laser perfusion imager review (V4.0, moor Instruments, UK) and the detailed data was also exported as txt format. Then the data were imported to the Matlab software and analyzed. For each recording point, the mean of blood flux for 20 min was calculated.

Previous studies indicated that blood flux oscillations at frequencies from 0.0095 to 1.6 Hz might reflect different physiological rhythms [13], which can be separated into five frequency bands in frequency domain [14–17]. In present study, wavelet analysis was performed on the blood flux signal (20 min) using a Morlet mother wavelet (The Mathworks Inc., Natick, MA, USA).

### 2.6. Coherence Analysis of Blood Flux Signals between Upper and Down Point

Coherence value (*C*_*xy*_(*f*)) can be used to examine the relation between two signals, and the analysis method can be referenced as in our previous study [18]. *C*_*xy*_(*f*) is between 0 and 1 and indicates how well *x* corresponds to *y* at each frequency. In present study, *x* is the upper point blood flux signal, while *y* is the down point blood flux signal. To calculate the coherence between upper point and down point, the coherence value was estimated by the following equation [19, 20]. The analysis was carried out with Matlab software.

(1) $$\begin{array}{c} C_{xy}\left(\;f\;\right)=\frac{{\left|P_{xy}\left(f\right)\right|}^{2}}{P_{xx}\left(f\right)P_{yy}\left(f\right)}. \end{array}$$

### 2.7. Statistical Analysis

Data are expressed as mean ± SE. The level of significance was defined as *P* < 0.05. Statistical analysis was performed by paired *t*-test with SPSS software (Version 13.0, SPSS Inc., Chicago, IL). All reported *P* values are two-sided.

## 3. Results

### 3.1. Mean Blood Flux at BL21

**Figure 2:**
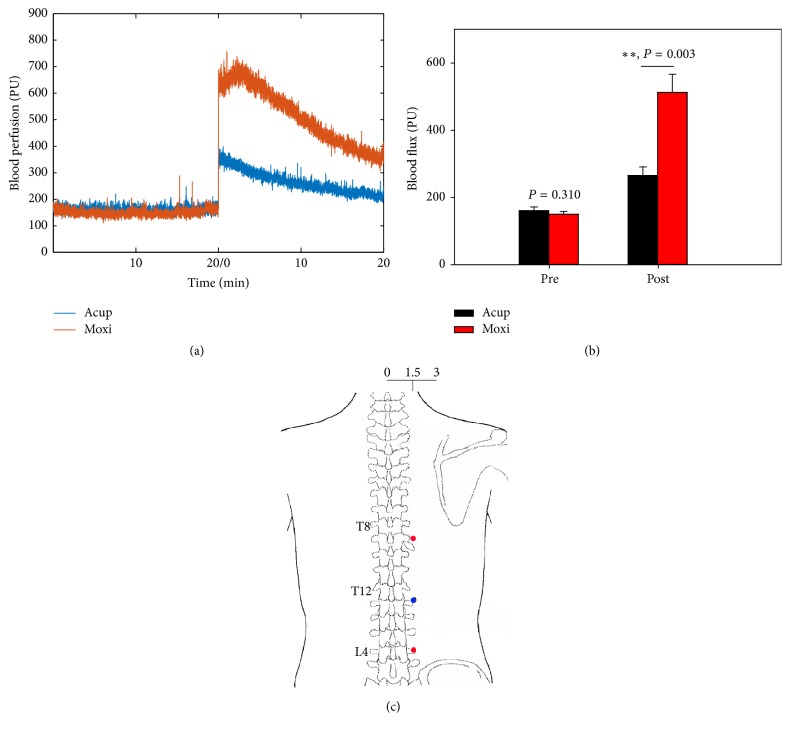
Original blood flux signals at right BL21 (a). Changes of skin blood flux at BL21 resulted from Acup or Moxi (b). ^*∗∗*^*P* < 0.01, Acup versus Moxi, paired* t*-test. All values are expressed as mean ± SE. Illustration of recording points (c), BL21 (blue) and upper point and down point (both in red).

The recording points (Figure 2(c)) and blood flux changes (Figure 2(a)) resulting from stimulation are shown in Figure 2, which indicated that after either type of stimulation, blood flux at BL21 was increased obviously. After Acup, the mean blood flux increased from 160.79 ± 10.82 PU during baseline to 265.66 ± 25.53 PU, while after Moxi the mean blood flux increased from 150.06 ± 8.29 PU to 512.67 ± 53.96 PU. Before stimulation, the mean blood perfusion has no significant difference (*t* = 1.076, *P* = 0.310, paired* t*-test), while after stimulation, the mean blood perfusion of BL21 has significant difference (*t* = −4.095, *P* = 0.003, paired *t*-test).

### 3.2. Wavelet Transform of Blood Flux at BL21

**Figure 3:**
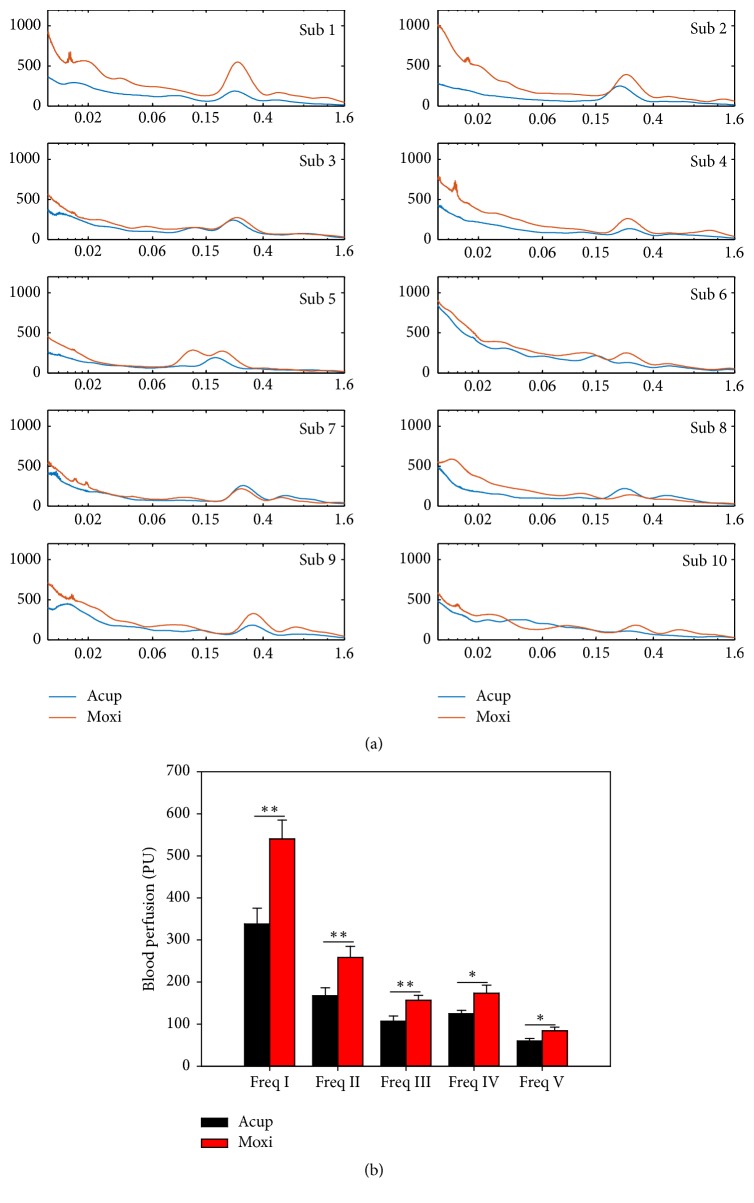
Morlet wavelet transform results. Case results (a) and compared results (b). ^*∗*^*P* < 0.05; ^*∗∗*^*P* < 0.01, Acup versus Moxi, paired* t*-test. All values are reported as mean ± SE.

To examine the underlying mechanisms after stimulation, blood flux signals were transformed by Morlet mother wavelet. There was significant difference between Acup and Moxi in the different frequency interval (Figure 3). In frequency interval I (0.0095–0.02 Hz), the blood perfusion was 338.06 ± 37.64 PU versus 540.35 ± 44.83 PU (*t* = −4.435, *P* = 0.002, paired* t*-test), respectively. In frequency interval II (0.02–0.06 Hz), the blood perfusion was 167.62 ± 18.71 PU versus 258.28 ± 26.48 PU (*t* = −4.004, *P* = 0.003, paired* t*-test), respectively. In frequency interval III (0.06–0.15 Hz), the blood perfusion was 107.16 ± 12.12 PU versus 156.54 ± 11.70 PU (*t* = −5.276, *P* = 0.001, paired* t*-test), respectively. In frequency interval IV (0.15–0.4 Hz), the blood perfusion was 124.98 ± 7.73 PU versus 173.83 ± 18.69 PU (*t* = −2.548, *P* = 0.031, paired* t*-test), respectively, and in frequency interval V (0.4–1.6 Hz), the blood perfusion was 59.96 ± 6.02 PU versus 84.27 ± 8.75 PU (*t* = −2.276, *P* = 0.049, paired* t*-test), respectively.

### 3.3. Wavelet Transform of Blood Flux at Upper Point and Down Point

**Figure 4:**
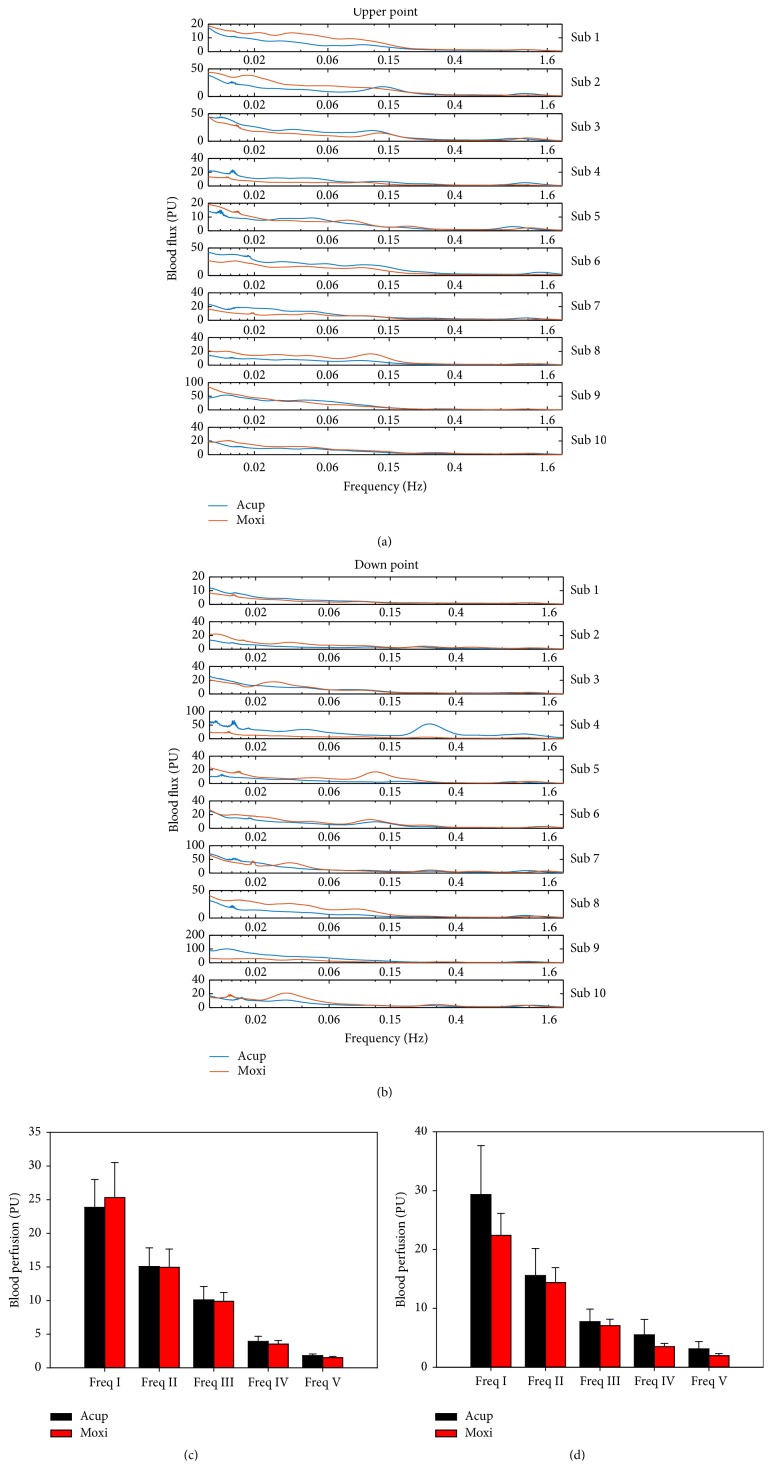
Morlet wavelet transform results of upper point and down point. Case results of upper point (a) and down point (b); compared results of upper point (c) and down point (d). All values are reported as mean ± SE.

There were no significant differences at the upper point (Figures 4(a) and 4(c)) resulting from Acup or Moxi stimulation. In frequency interval I (0.0095–0.02 Hz), the blood perfusion was 23.85 ± 4.14 PU versus 25.30 ± 5.21 PU (*t* = −0.480, *P* = 0.643, paired* t*-test), respectively. In frequency interval II (0.02–0.06 Hz), the blood perfusion was 15.05 ± 2.79 PU versus 14.96 ± 2.70 PU (*t* = 0.45, *P* = 0.965, paired* t*-test), respectively. In frequency interval III (0.06–0.15 Hz), the blood perfusion was 10.09 ± 2.00 PU versus 8.89 ± 1.32 PU (*t* = 0.130, *P* = 0.9, paired* t*-test), respectively. In frequency interval IV (0.15–0.4 Hz), the blood perfusion was 3.92 ± 0.76 PU versus 3.54 ± 0.53 PU (*t* = 0.676, *P* = 0.516, paired* t*-test), respectively, and in frequency interval V (0.4–1.6 Hz), the blood perfusion was 1.83 ± 0.23 PU versus 1.52 ± 0.18 PU (*t* = 1.485, *P* = 0.172, paired* t*-test), respectively.

There were also no significant differences at down point (Figures 4(b) and 4(d)) resulting from Acup or Moxi stimulation. In frequency interval I (0.0095–0.02 Hz), the blood perfusion was 29.35 ± 8.29 PU versus 22.41 ± 3.73 PU (*t* = 1.022, *P* = 0.334, paired* t*-test), respectively. In frequency interval II (0.02–0.06 Hz), the blood perfusion was 15.56 ± 4.62 PU versus 14.39 ± 2.51 PU (*t* = 0.298, *P* = 0.772, paired* t*-test), respectively. In frequency interval III (0.06–0.15 Hz), the blood perfusion was 7.73 ± 2.14 PU versus 7.07 ± 1.09 PU (*t* = 0.289, *P* = 0.779, paired* t*-test), respectively. In frequency interval IV (0.15–0.4 Hz), the blood perfusion was 5.51 ± 2.59 PU versus 3.49 ± 0.53 PU (*t* = 0.797, *P* = 0.446, paired* t*-test), respectively, and in frequency interval V (0.4–1.6 Hz), the blood perfusion was 3.11 ± 1.25 PU versus 1.96 ± 0.36 PU (*t* = 0.995, *P* = 0.346, paired* t*-test), respectively.

### 3.4. Coherence Analysis Result between Upper Point and Down Point

**Figure 5:**
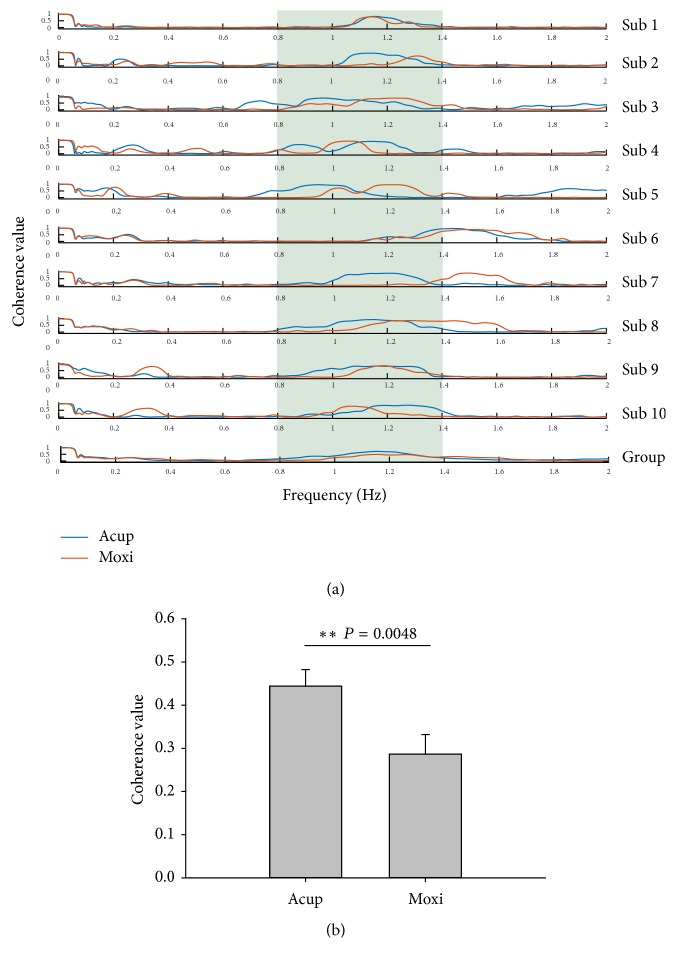
Coherence value of blood perfusion between upper point and down point. Case results (a) and compared results of mean coherence value from 0.8 Hz to 1.4 Hz frequency interval (b). ^*∗∗*^*P* < 0.01, Acup versus Moxi. All values are reported as mean ± SE.

The coherence-frequency responses to stimuli are shown in Figure 5. In the frequency-coherence curve, there is a very clear peak from 0.8 to 1.4 Hz and significant differences of mean coherence value in this frequency interval were observed (0.44 ± 0.04 versus 0.29 ± 0.06, *t* = 3.710, *P* = 0.0048, paired* t*-test).

## 4. Discussion

The main finding of this study is that there have been different responses of blood perfusion at BL21 acupoint after Acup or Moxi stimulation. To the best of our knowledge, the current pilot study is the first to compare vascular response after Acup-related stimulations by Morlet wavelet analysis. Whereas other studies have detected changes in local vascular response [21, 22], this study is the first to detect blood flux signals coherence along BL meridian. From our study, Acup and Moxi not only affect the average blood flux of regional BL21 in different pattern, but also have the different effect on coherence along BL meridian.

Previous studies indicated that blood flux oscillations at frequencies from 0.0095 to 1.6 Hz might reflect different physiological rhythms [13], which can be separated into five frequency bands in frequency domain [14–17]. In the present study, the Morlet mother wavelet transform was used to separate frequency interval and this method provided a noninvasive access to examine the mechanisms of blood flow regulation. From current results, all components of blood perfusion signals participated in the local blood regulation, which indicated that the response to a special stimulation at acupoint is a comprehensive process. The different response patterns of blood flow can partly explain the heterogeneity between Acup and Moxi.

In Acup theory, the Acup effect is based on the integrity function of the meridian [23]. Some studies suggested that the meridian system may contain a continuous channel [9] to facilitate signal transport in peripheral tissues [10, 11]. According to this view, the vascular response along the meridian has intrinsic relevance. However, so far, there is the still lack of effective approaches to evaluate this intrinsic relevance.

Coherence analysis is a method to estimate the correlation of the signals on different frequencies, which can be used for analysis correlation of two blood flux signals in frequency domain [24–26]. In the previous study, the coherence analysis along PC meridian was carried out in different aged human before and after 30 mmHg inflating occlusion [27]. Further study indicated that coherence analysis might be used to assess microcirculatory changes at different ages [28] and oral load different cold water [29]. In the current study, the coherence-frequency curves were significantly different after stimulation in the frequency band of 0.8–1.4 Hz, suggesting that Acup and Moxi have different effects on the coherence of BL meridian. Therefore, we believed that the coherence analysis might be a potential method which can be used to analyze this intrinsic relevance of blood flux along the meridian.

## 5. Conclusion

There have been different responses of blood perfusion at BL21 acupoint after Acup or Moxi stimulation. Coherence analysis along BL meridian can also as be an appropriate index to distinguish different stimulations.

## Figures and Tables

**Table 1:** Subject's individual characteristics.

Subject number	M/F	Age (year)	Height (cm)	Weight (kg)	BMI
Sub 1	M	32	172	85	28.73
Sub 2	M	38	172	80	27.04
Sub 3	F	26	164	47	17.47
Sub 4	F	27	160	45	17.58
Sub 5	F	26	164	53	19.71
Sub 6	M	27	168	68	24.09
Sub 7	F	28	158	44	17.63
Sub 8	F	24	154	46	19.40
Sub 9	M	25	170	60	20.76
Sub 10	F	27	169	65	22.76
Mean ± SD	4M/6F	28 ± 4	165.10 ± 6.20	59.30 ± 14.92	21.52 ± 4.03

## Abbreviations

FLPI: Full-field laser speckle perfusion imager
EA: Electroacupuncture
Acup: Acupuncture
Moxi: Moxibustion
BL21: Weishu acupoint
BL meridian: Bladder meridian
PC meridian: Pericardium meridian
TCM: Traditional Chinese Medicine
BMI: Body mass index
Fig: Figure
SD: Standard deviation
SE: Standard error.
